# Inhibitory control towards angry stimuli in patients with binge eating disorder: a pilot study

**DOI:** 10.1186/s40337-023-00848-2

**Published:** 2023-07-31

**Authors:** Kathrin Schag, Lea Sandler, Stephan Zipfel, Birgit Derntl, Katrin Elisabeth Giel

**Affiliations:** 1 Department of Psychosomatic Medicine and Psychotherapy, Medical University Hospital Tübingen, Osianderstraße 5, 72076 Tübingen, Germany; 2 Centre of Excellence for Eating Disorders Tübingen (KOMET), Tübingen, Germany; 3 DZPG (German Center for Mental Health), Tübingen, Germany; 4 Department for Psychiatry and Psychotherapy, Tübingen Center for Mental Health (TüCMH), University of Tübingen, Tübingen, Germany

**Keywords:** Anger, Binge eating disorder, Eating disorder, Emotion, Impulsivity, Inhibitory control, Mood, Stop signal task

## Abstract

**Background:**

Emotion regulation theories and the negative urgency concept assume that negative mood increases binge eating. Negative emotions are considered as a trigger for binge eating, while binge eating itself is regarded as an impulsive behavior and should thus be increased within the negative urgency concept. Anger might be a specific negative emotion triggering binge eating in patients with binge eating disorder (BED). We investigated how inhibitory control as one main factor of impulsivity is influenced by anger stimuli in patients with BED and two control groups.

**Methods:**

We compared patients with BED (N = 20) with normal-weight healthy control participants (NW-CG, N = 20) and BMI-matched overweight and obese control participants (BMI-CG, N = 18). We used the emotional Stop Signal task (eSST) to investigate inhibitory control, where we presented angry facial expressions in comparison with neutral facial expressions as emotional stimuli.

**Results:**

All participants showed decreased inhibitory control in the angry versus neutral condition, i.e., a faster Stop Signal Reaction Time and a lower percentage of correct reactions. However, no significant group differences emerged in terms of performance. Performance in the eSST did not correlate with negative urgency, disorder- or emotion-related characteristics.

**Conclusions:**

The current pilot study does not deliver evidence for decreased inhibitory control towards angry stimuli in patients with BED, as we detected a general and not disorder-related effect in all participants that might represent the conjunction of inhibitory control and anger. A direct mood induction technique might have led to different results. Further research in healthy and clinical groups is needed.

## Abstract (plain language summary)

Several theories suggest that binge eating episodes are often appearing in negative mood and anger is one specific emotion that might commonly trigger binge eating. The aim of this study was to investigate inhibitory control under anger in patients with binge eating disorder (BED). Inhibitory control is a specific component of impulsivity and further studies suggest that inhibitory control deficits are related to binge eating. We compared patients with BED with normal-weight participants and overweight or obese participants as control groups in a specific inhibitory control task, where faces with angry or neutral expressions were presented and participants should stop, i.e. inhibit their reactions. All participants had more inhibitory control deficits when they viewed angry faces in comparison with neutral expressions, and patients with BED did not differ from the participants of the two control groups. Thus, all participants react on anger with increased impulsivity indicating that patients with BED seem to have no specific deficit in this regard. As this was a pilot study, further studies should investigate the interplay of several emotions and impulsivity in healthy and clinical groups.

## Background

Binge Eating Disorder (BED) is characterized by recurrent binge eating episodes, i.e., eating large amounts of food in a discrete period of time while experiencing loss of control [1]. Current neurobiological models of BED describe the pivotal role of inhibitory control, reward processing and emotion regulation in the etiology and maintenance of this eating disorder [2–5]. For example, several theoretical models [5–7] postulate that negative emotions might trigger binge eating episodes, and that binge eating might act as a dysfunctional emotion regulation strategy. Reviews and meta-analyses have synthesized evidence at least for the first part of these models, i.e., the trigger function of negative mood for binge eating in patients with BED [5, 8–10]. However, evidence on effects of several negative emotions have been pooled in these reviews [5, 8, 9] and it has not yet been disentangled, if there are differences between discrete negative emotions. In their systematic review, Nicholls et al. [10] report that evidence concerning anger as a trigger for binge eating is sparse and heterogenous as most previous studies investigated sad mood. Therefore, it was the aim of the current study to investigate the impact of one specific negative emotion on information processing in patients with BED. Specifically, we decided to investigate anger as patients with BED report most often feelings of anger preceding a binge eating episode [11]. Moreover, patients with BED indicated increased emotional eating compared to patients with Anorexia Nervosa [12] or a stronger desire to eat compared to obese and normal-weight controls [11] in response to discrete negative emotions including anger. However, to our knowledge, there is so far no laboratory study that investigated the impact of anger on information processing as a potential underlying mechanism of binge eating. At least, first studies imply that sad mood might impact information processing in patients with BED (e.g. [13, 14]). Apart from the emotion regulation theories in BED mentioned above [5–7], there is a more general concept from the four factor model of impulsivity [15, 16], the negative urgency concept. It postulates that impulsive behavior is more common in negative mood [16]. Thus both, the emotion regulation theories [5–7] as well as the negative urgency concept [15, 16] assume that binge eating is increased in negative mood, the former considering negative emotions as a trigger specifically for binge eating, the latter if binge eating is regarded as an impulsive behavior. In line with the negative urgency concept, the review by Heatherton and Wagner [17] reports evidence from neuroimaging studies that negative emotions impair the inhibitory function of prefrontal cortex (PFC) areas in favor of ventral regions involved in reward and emotion processing, e.g., regarding the regulation of eating and addictive behaviors. Thus, anger might foster binge eating by downregulating inhibitory control of PFC over ventral regions, i.e., patients with BED might have difficulties to inhibit or stop eating. Indeed, neuroimaging, behavioral and self-report studies summarized in several reviews [3, 4, 18–21] indicate that increased impulsivity is one promising underlying mechanism for binge eating, especially concerning the impulsivity components negative urgency and inhibitory control. For example, Giel et al. [3] systematically reviewed studies using inhibitory control tasks like the Go-NoGo or Stop Signal task that were modified with food stimuli and reported decreased inhibitory control in patients with BED compared with obese and normal-weight controls. Fischer et al. [19] report in their meta-analysis based on 50 self-report studies that negative urgency is most related to bulimic symptoms (weighted *r* = 0.38) compared to other impulsivity constructs like lack of planning or lack of persistence. Regarding the impact of negative emotions, students with high negative urgency and high arousal after anger induction ate more sweets than fruits [22]. Moreover, adolescents with low inhibitory control experienced more loss of control eating at a multi-item test meal buffet in sad versus neutral mood [23]. However, negative urgency and inhibitory control in these two studies have been classified by self-reports in nonclinical samples and the impact of negative emotions on inhibitory control in patients with BED has not yet been investigated.

To investigate inhibitory control, we have chosen a widely used laboratory task, the Stop Signal task (SST) [24]. Patients with BED have shown decreased inhibitory control in comparison with healthy normal-weight controls in the SST, but evidence was mixed in comparison with obese patients without BED [25, 26], which might be due to the impact of uncontrolled current mood [27]. Clearer results have been delivered, if disorder-related stimuli like food have been presented while executing inhibitory control tasks [3, 18]. Therefore, stimuli with high emotional valence presenting food or negative emotions might decrease inhibitory control in the SST in patients with BED. One possible explanation of this might be that angry facial expressions impair the PFC by the activation of limbic regions as self-regulation capacities are limited in concordance with the self-regulation model of Heatherton and Wagner [17]. In line, a current fMRI study in healthy participants delivered evidence that angry facial stimuli activate a widespread neural observation/execution network including the amygdala besides frontal areas including the precentral gyrus, right pallidum, several regions in the cerebellum, and brainstem [28]. Another general population study using angry versus happy faces as the stop signal in the SST indicated that angry faces impair inhibitory control as they capture more attention compared to happy faces [29]. Moreover, according to the self-regulation model of Heatherton and Wagner [17], emotional stimuli facilitate limbic bottom-up processes and inhibit top-down self-regulatory processes, especially in patients with mental disorders as indicated by neuroimaging studies where patients show impaired prefrontal functioning and reduced connectivity between the PFC and the amygdala.

Thus, we investigated in the current pilot project, if patients with BED show decreased inhibitory control in a modified version of the SST, i.e. the emotional Stop Signal task (eSST) that has been developed by our workgroup and used in previous studies with clinical samples [30]. In the eSST, angry facial expressions as a specific negative emotion are presented as stimuli in comparison with facial control stimuli with a neutral expression. We compared eSST performance in patients with BED with normal-weight healthy control participants (NW-CG) and BMI-matched overweight and obese control participants (BMI-CG) to disentangle potential mechanisms related to BED and obesity [27].

In particular, we hypothesized that [1] all participants will show decreased inhibitory control in the condition with angry versus neutral facial expressions in accordance with the general negative urgency concept [16] and the self-regulation model of Heatherton and Wagner [17]. Moreover, we suggest that [2] patients with BED will show decreased inhibitory control in comparison with two control groups in the eSST in both stimuli conditions, i.e. while presenting angry and neutral facial expressions, but most prominent in the condition with angry versus neutral facial expressions [5, 27]. Due to the evidence related to obesity [3, 18, 25, 26], we hypothesized that [3] the BMI-CG will perform better than patients with BED in the eSST, but lower than the NW-CG. Furthermore, we explored [4] if inhibitory control is related to disorder-related, impulsive or emotion-related characteristics of the sample.

## Methods

### Participants

Participants were 20 female adults with BED according to DSM-5 [1] in the BED group, 18 age- (± 5 years) and weight-matched (± 5 kg/m^2^) females in the BMI-CG, and 20 age-matched females with normal weight (18.5–24.9 kg/m^2^) in the NW-CG. They were recruited by emails, flyers and from the treatment services of the Department of Psychosomatic Medicine and Psychotherapy, Tübingen, Germany.

Participants were excluded if they were currently pregnant, breast-feeding, suicidal or reported a neurological or somatic disorder as well as medication or medical interventions influencing the central nervous system, eating behavior or weight. They were further excluded if they fulfilled DSM-5 criteria for a current moderate / severe substance use disorder, a psychotic disorder or a bipolar I disorder. Additionally, participants from the BMI-CG and NW-CG were excluded if they presented any current mental disorder besides a specific phobia.

### ***Emotional stop signal task ***(eSST; [30]) ***and stimulus material ***[31]

**Fig. 1:**
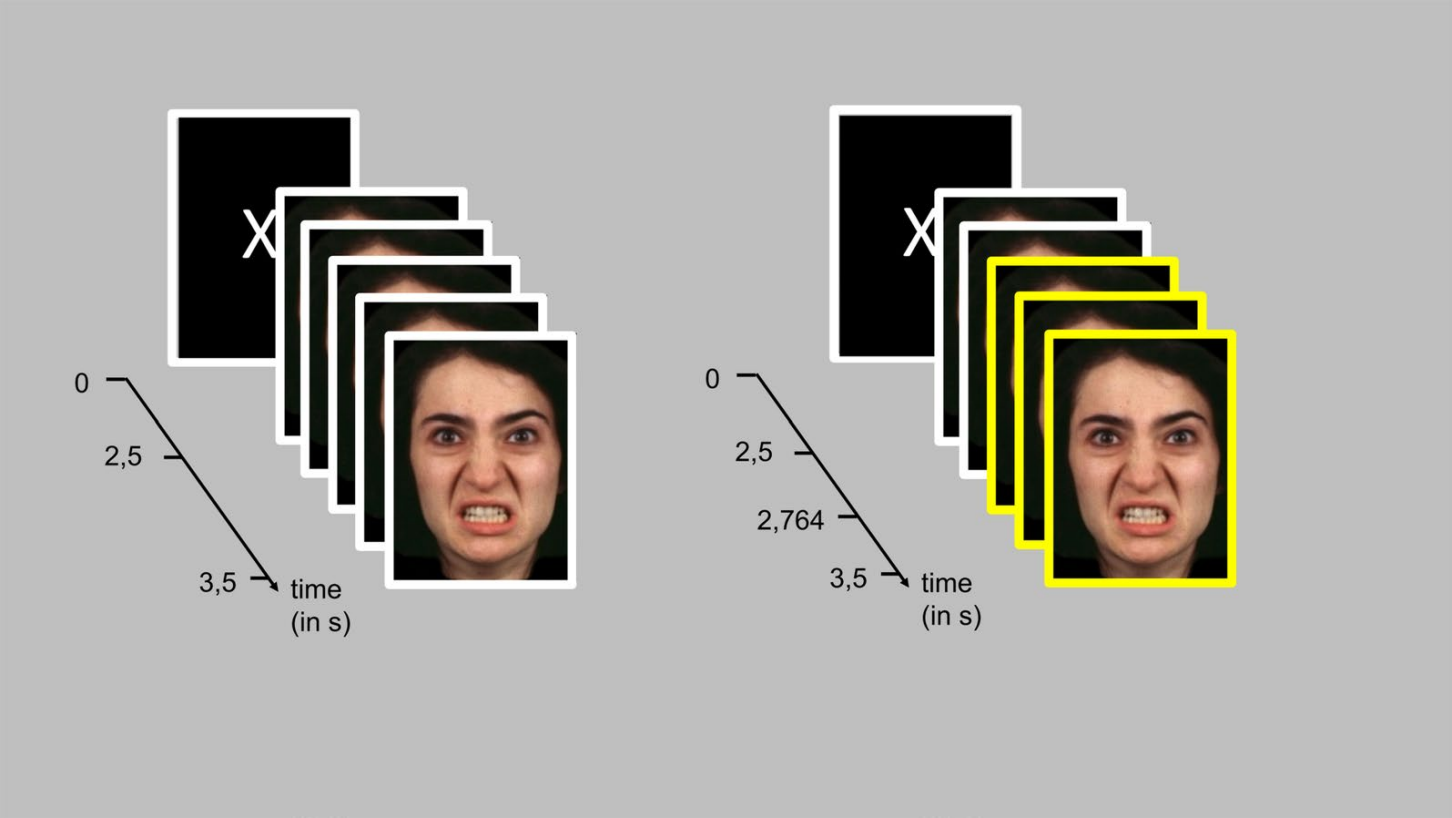
Exemplary trials of the emotional Stop Signal Task (eSST). *Legend* One go trial on the left side and one stop trial on the right side of the emotional Stop Signal Task with an angry stimulus according to the Brain Behavior Laboratory of the University of Pennsylvania [31]

The eSST [30] measures the ability to inhibit impulsive reactions towards emotional stimuli and has been adapted from the classical paradigm [24]. The basic task of the participant is to respond to one simple stimulus like a light or a geometric form, but not to respond when another stimulus, the stop signal, occurs several milliseconds later [24]. We used stimulus material from the Brain Behavior Laboratory of the University of Pennsylvania [31] consisting of 120 different emotional expressions as performed by actors. These depicted either angry or neutral facial expressions, 50% were performed by female faces and 50% by male faces. Before each trial, a fixation cross was displayed for 2.5–3.5 s in random order (see Fig. 1). In go trials, participants were instructed to press as fast as possible the space button of a Dell Vostro 1720 notebook upon stimulus presentation. In stop trials, which were indicated by a yellow frame (stop signal) around the picture, participants were instructed to withhold the button press. The latency of the stop signal was adaptive. It started at 200 ms after presentation of the first stimulus and was then adapted according to the performance of each participant in the previous trial (± 64 ms). We presented 400 trials in randomized order in 3 blocks, where 75% were go trials and 25% were stop trials, and where 50% were angry and 50% were neutral facial stimuli. As outcomes, we computed (a) the Stop Signal Reaction Time (SSRT) that is the mean reaction time in go trials relative to the latency of the stop signal, and (b) the percent of correct reactions in go and stop trials.

### ***Vienna emotion recognition task (VERT-K; ***[32]***)***

The VERT-K investigates the ability to correctly recognize the different emotional expressions from stimuli of the Brain Behavior Laboratory of the University of Pennsylvania [31]. It consists of 36 trials displaying facial expressions of the five basic emotions anger, disgust, fear, happiness and sadness as well as six neutral facial expressions. Stimuli are balanced for sex of poser and were presented once in randomized order. Participants were instructed to choose spontaneously the appropriate emotion by button press out of six possible answers. The VERT-K was used in this study to control, if the emotional expressions were correctly identified by the participants.

### ***Self-assessment manikins (SAM; ***[33]***)***

The SAM were used to assess current mood of the participants. The SAM represent mood on the three dimensions valence, arousal and dominance. The dimension valence represents the extent of feeling a positive versus negative emotion, the dimension arousal represents the extent of feeling active or excited, and the dimension dominance represents the extent of feeling control or efficacy. For each dimension, the participants had to choose the currently most appropriate manikin out of five options that display different levels of each dimension, so that 1 represents negative mood, low arousal or low dominance, whereas 5 represents positive mood, high arousal or high dominance.

### Procedure

After declaring written informed consent, sociodemographic variables were assessed and height and weight were measured to compute BMI. Then, the eSST was performed. Current mood was assessed with SAM immediately before and after eSST. Afterwards, the Structured Clinical Interview for mental disorders (SCID-I; [34]) was used to diagnose BED as well as comorbid mental disorders. Then, the VERT-K [32] was conducted. Finally, the participants filled in several validated self-report instruments, i.e. the Eating Disorder Examination Questionnaire (EDE-Q; [35]) to assess eating disorder pathology, the UPPS Impulsive Behavior Scale (UPPS; [36]) to assess impulsivity, the Generalized expectancies for negative mood regulation questionnaire (NMR-SF; [37]) to assess emotion regulation, the Becks’ Depression Inventory II (BDI-II; [38]) to assess severity of depressive symptoms, and the Trait scale of the State-Trait-Anger-Inventory-2 (STAXI-2; [39]).

### Statistical analysis

All data was analyzed with the Statistical Package for the Social Sciences, Version 24. Sample characteristics were analyzed with appropriate parametric or nonparametric tests with the between-subjects factor group (BED; BMI-CG; NW-CG) at an alpha level of *p* = 0.05 and pairwise Bonferroni corrected post hoc comparisons after imputation with EM algorithm of single missings (14 missings out of 160 items in 58 patients, i.e., < 1%). Variables of the manipulation check (VERT-K, SAM) were not normally distributed, so that they were analyzed with Kruskal–Wallis tests with the between-subjects factor group, Friedman test with the within-subjects factor emotion and post-hoc Dunn-Bonferroni tests for pairwise comparisons (VERT-K, 6 emotions) or Wilcoxon signed ranks tests with the within-subjects factor measurement point (SAM, pre vs. post eSST). Regarding eSST, five participants were excluded from data analysis, because they deviated more than three interquartiles from the mean of correct reactions (%) which indicates an inappropriate processing of the task. Thus, 18 participants of the BED group, 16 of the BMI-CG and 19 of the NW-CG were analyzed. As eSST outcomes were not normally distributed, they were analyzed with Kruskal–Wallis tests or Wilcoxon signed ranks tests with the between-subjects factor group or the within-subjects factor stimuli (angry vs. neutral). The difference of angry—neutral stimuli was used as outcome to examine group × stimulus interactions. For pairwise comparisons, post-hoc Dunn-Bonferroni tests were applied. Additionally, Spearman correlations between eSST (SSRT and correct reactions concerning angry stimuli) and EDE-Q total score, UPPS negative urgency, NMR-SF total score, VERT-K anger and neutral scores (correct answers %), STAXI-2 Trait anger total score, BMI as well as BDI II total score were computed and Bonferroni corrected to a significance level of *p* = 0.003.

## Results

### Sample characteristics

An overview of the sample characteristics is presented in Table 1. Further, 53 (91.4%) of the participants were German, 36 (62.1%) of the participants were singles, 13 (22.4%) married and 9 (15.5%) divorced. Concerning school education, the groups did not differ from each other and reported a high education level (*M*_*overall*_ = 12.36 years, *SD*_*overall*_ = 1.35; *H*(2) = 1.92, *p* = 0.384). Concerning mental disorders, 9 current comorbid mental disorders in the BED group were reported (3 affective disorders, 5 anxiety disorders, 1 somatoform disorders), 2 specific phobias in the BMI-CG and 1 in the NW-CG.

**Table 1:** Sample characteristics of the BED, BMI-CG and NW-CG group

	BED (N = 20) *M (SD)*	BMI-CG *(N* = *18)* *M (SD)*	NW-CG *(N* = *20)* *M (SD)*	*Test statistics*	Overall *p* value	*p* value BED versus BMI-CG	*p* value BED versus NW-CG	*p* value BMI-CG versus NW-CG
Age	37.0 (14.1)	36.6 (13.9)	36.5 (13.8)	.023^*b*^	.989	–	–	–
BMI (kg/m^2^)	35.2 (11.2)	32.9 (9.8)	21.9 (1.5)	13.74^*a*^	< .001*	.770	< .001*	< .001*
EDE-Q total	3.0 (1.0)	1.1 (1.0)	0.4 (0.4)	34.36^*b*^	< .001*	.001*	< .001*	.119
BDI-II total	15.7 (12.6)	3.7 (6.4)	3.2 (3.1)	20.92^*b*^	< .001*	< .001*	< .001*	1.00
NMR-SF total	47.9 (10.0)	54.1 (10.9)	59.6 (6.1)	8.24^*a*^	.001*	.178	< .001*	.157
UPPS negative urgency	34.0 (4.9)	24.4 (4.9)	22.5 (4.3)	34.17^*a*^	< .001*	< .001*	< .001*	.479
UPPS premediation	23.2 (5.5)	24.1 (4.2)	22.4 (3.4)	.69^*a*^	.506	–	–	–
UPPS perseverance	23.4 (4.4)	20.3 (5.3)	17.9 (3.3)	8.04^*a*^	.001*	.102	.001*	.236
UPPS sensation seeking	35.3 (7.4)	30.7 (7.4)	29.9 (8.3)	2.86^*a*^	.066	–	–	–
STAXI-2 Trait anger total	22.2 (3.6)	20.7 (5.3)	19.1 (3.9)	2.73^*a*^	.074	–	–	–

* The level of significance is *p* < .05

One-factorial variance analyses^a^ or Kruskal–Wallis Tests^b^ with overall tests as well as pairwise Bonferroni-corrected post hoc comparisons

*BDI II* Becks Depression Inventory II, *BMI* Body Mass Index, *EDE-Q* Eating Disorder Examination Questionnaire, *NMR-SF* Generalized expectancies for negative mood regulation questionnaire, *STAXI-2* State-Trait-Anger-Inventory-2

### Manipulation check

Correct answers (%) from the VERT-K imply that all participants were able to correctly identify the emotional expressions, in particular angry and neutral stimuli (see Table 2). Groups did not differ from each other [*H*(2) = 0.48, *p* = 0.787], but the kind of emotion affected the results [*χ*^2^(5) = 130, *p* < 0.001]: Happiness was more often correctly identified as compared to anxiety, sadness and disgust, whereas happiness, anger and neutral expressions did not differ from each other (for details see Table 2).

**Table 2:** Emotion perception in the VERT-K (Vienna Emotion Recognition Task)

	M (SD)	*p* values of Bonferroni-corrected pairwise comparisons					
	Correct answers (%)	Happiness	Neutral	Anger	Anxiety	Sadness	Disgust
Happiness	96.6 (7.5)	–	1.0	.148	< .001*	< .001*	< .001*
Neutral	90.8 (13.4)	–	–	1.0	.103	< .001*	< .001*
Anger	89.1 (11.5)	–	–	–	1.0	< .001*	< .001*
Anxiety	79.3 (23.0)	–	–	–	–	.103	< .001*
Sadness	71.3 (7.9)	–	–	–	–	–	1.0
Disgust	61.2 (17.2)	–	–	–	–	–	–

* The level of significance is *p* < .05

Correct answers (%) are presented for each discrete emotion in descending order and pooled over the groups BED, BMI-CG and NW-CG (*N* = 58)

Concerning current mood, SAM pre and post values did not differ between groups concerning arousal and dominance (all *p* > 0.05), but concerning valence (see Table 3). Before eSST, the BED group reported lower mood compared with the NW-CG, whereas after eSST, no significant differences in pairwise comparisons occurred. Valence pre and post data did not correlate with the eSST outcomes [pre: SSRT *r* = 0.08, *p* = 0.592; correct reactions (%) *r* =  − 0.13, *p* = 0.362, post: SSRT *r* =  − 0.12, *p* = 0.394; correct reactions (%) *r* = 0.07, *p* = 0.597], so that we did not include them as a covariate into the eSST analyses.

**Table 3:** Mood ratings with Self Assessment Manikins (SAM) before (pre) and after (post) eSST

	BED (N = 20) *M (SD)*	BMI-CG *(N* = *18)* *M (SD)*	NW-CG *(N* = *20)* *M (SD)*	M (SD) Pooled over groups (N=58)	Test statistic H	Overall *p* value	*p* value BED versus BMI-CG	*p* value BED versus NW-CG	*p* value BMI-CG versus NW-CG
Pre valence	3.8 (0.6)	4.2 (0.6)	4.7 (0.5)	4.2 (0.7)	17.84	< .001*	–	< .001*	–
Post valence	3.7 (0.7)	3.7 (0.9)	4.2 (0.6)	3.9 (0.8)	6.24	.044*	–	–	–
Pre arousal	2.6 (0.8)	2.8 (0.7)	2.7 (0.6)	2.7 (0.7)	1.04	.594	–	–	–
Post arousal	2.4 (0.8)	2.2 (0.6)	2.3 (0.7)	2.3 (0.7)	0.67	.715	–	–	–
Pre dominance	3.1 (0.7)	3.3 (0.5)	3.2 (0.4)	3.2 (0.5)	1.32	.516	–	–	–
Post dominance	2.9 (0.7)	3.1 (0.5)	3.1 (0.4)	3.0 (0.6)	1.96	.376	–	–	–

* The level of significance is *p* < .05

Kruskal–Wallis Tests with overall tests as well as pairwise Bonferroni-corrected posthoc comparisons

Over time, SAM values were reduced after eSST compared with before eSST over all groups at all three dimensions (valence *z* =  − 3.41, *p* < 0.001; arousal *z* =  − 3.45, *p* < 0.001; dominance *z* =  − 2.14, *p* = 0.033). Pre-post difference scores yielded no significant group differences (all *p* > 0.05), i.e. no interactional effects occurred.

### Inhibitory control towards emotional stimuli

**Fig. 2:**
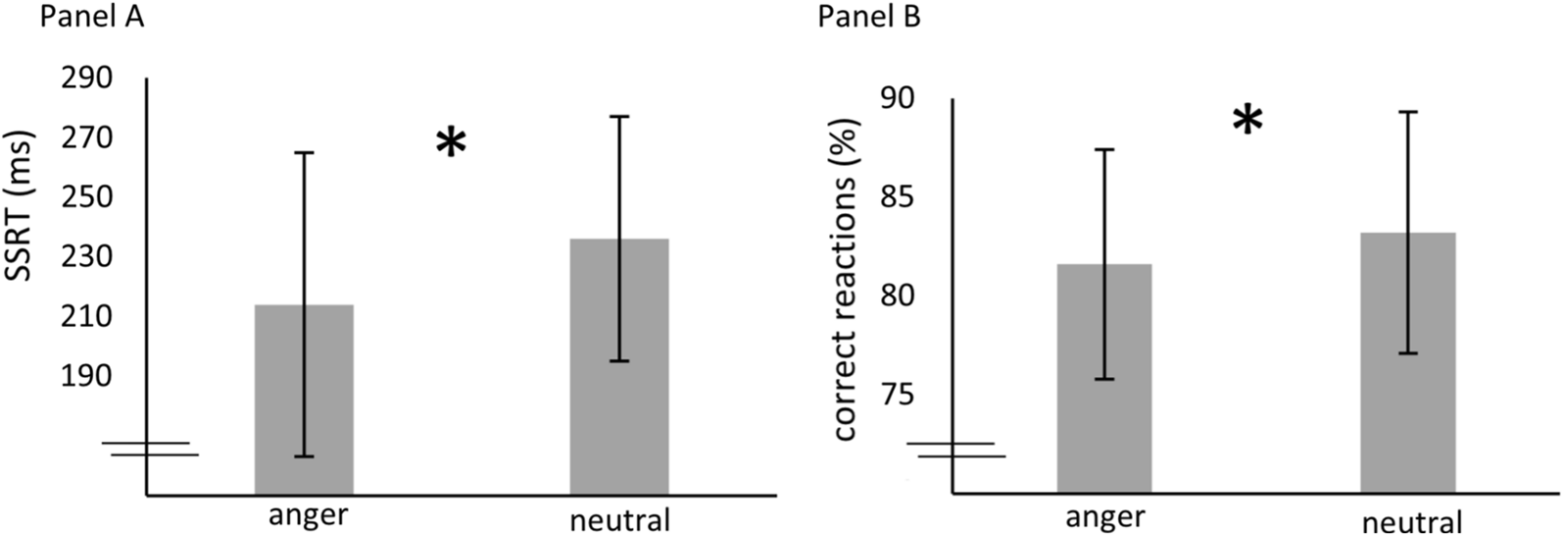
Performance in the eSST (emotional Stop Signal Task). *Legend.* Panel A: SSRT (Stop Signal Reaction Time) and Panel B: Correct reactions (%) of the for angry versus neutral stimuli, pooled over the groups BED, BMI-CG and NW-CG. Significant differences are indicated with*

As displayed in Table 4, there was no significant group effect regarding SSRT [*H*(2) = 1.85, *p* = 0.397] and correct reactions (%) [*H*(2) = 1.47, *p* = 0.480] in the eSST. However, according to Fig. 2, there was a main effect of emotion (*z* = *4.98*, *p* < 0.001), indicating significantly shorter SSRT towards angry than neutral stimuli across all groups, and also in terms of accuracy (*z* = 3.07, *p* = 0.002), as participants made more errors concerning angry than neutral stimuli. No group-by-emotion interaction reached significance (all *p* > 0.05).

**Table 4:** Descriptive statistics (*M, SD*) of the ESST (emotional Stop Signal Task)

	BED (N = 18)	BMI-CG (N = 16)	NW-CG (N = 19)	*M (SD)* Pooled over groups
	M (SD)	M (SD)	M (SD)	(N = 53)
*SSRT (ms)*				
Overall	220 (39)	241 (52)	217 (39)	225 (44)
Anger	212 (45)	230 (62)	204 (44)	214 (51)
Neutral	229 (37)	251 (48)	230 (38)	236 (41)
*Correct reactions (%)*				
Overall	83.6 (4.6)	81.9 (6.6)	81.7 (5.8)	82.4 (5.7)
Anger	82.9 (4.3)	80.9 (6.7)	81.0 (6.2)	81.6 (5.8)
Neutral	84.3 (5.7)	82.9 (7.0)	82.4 (5.8)	83.2 (6.1)

SSRT (Stop Signal Reaction Time) and correct reactions (%) are presented separately for the groups BED, BMI-CG and NW-CG and for angry versus neutral stimuli

### Correlational analyses

SSRT and correct reactions (%) towards angry stimuli in the eSST did not correlate with any of the explored variables (see Table 5).

**Table 5:** Correlational analyses between eSST outcomes towards angry stimuli and disorder-related, impulsive or emotion-related characteristics

		UPPS negative urgency	NMR-SF total	STAXI-2 Trait anger total	EDE-Q total	BMI (kg/m^2^)	BDI-II total	VERT-K anger correct (%)	VERT-K neutral correct (%)
SSRT anger	*r*	− .07	.12	− .02	− .12	.15	− .17	.20	.05
	*p*	.608	.396	.896	.378	.292	.229	.161	.718
Correct reactions (%) anger	*r*	− .04	− .05	− .14	.03	− .17	− .002	.13	.07
	*p*	.761	.723	.341	.821	.223	.988	.343	.599

Significance level is Bonferroni-corrected to *p* < .003

*BDI II* Becks Depression Inventory II, *BMI* Body Mass Index, *EDE-Q* Eating Disorder Examination Questionnaire, *NMR-SF* Generalized expectancies for negative mood regulation questionnaire, *STAXI-2* State-Trait-Anger-Inventory-2

## Discussion

The current pilot study investigated whether inhibitory control is especially decreased in patients with BED when angry versus neutral facial expressions are presented. Data were compared with a BMI-matched control group (BMI-CG) as well as a normal weight control group (NW-CG) in a well characterized sample, while controlling for potential confounders like emotion perception and current mood, and by using an objective and validated laboratory task, the emotional Stop Signal Task (eSST). Like expected in hypothesis (1), all participants showed decreased inhibitory control in the angry versus neutral condition, i.e., a faster SSRT and less correct reactions (%). Against our hypothesis (2), patients with BED did not show decreased inhibitory control in the eSST compared with the BMI-CG and the NW-CG, neither in the angry nor in the neutral condition. BMI-CG and NW-CG did also not differ from each other (hypothesis 3). Correlational analyses (4) yielded no significant associations between the eSST and negative urgency, disorder- or emotion-related sample characteristics.

The main effect concerning stimulus condition implies that all participants had decreased inhibitory control when angry versus neutral facial expressions have been presented. Based on these results, we cautiously hypothesize that the angry stimuli in the eSST might have been able to impair the PFC in favor of limbic regions like the amygdala, which would be in line with the self-regulation model postulated by Heatherton and Wagner [17]. This speaks for a general negative urgency effect in the eSST that concerns all participants, irrespective of eating behavior. It might be that this effect was so strong that it overrides a putative eating-disorder related effect in patients with BED. Additionally, as we have not included other negative facial expressions, it remains unclear if our results concerning decreased inhibitory control are specific for anger.

According to the evidence from several reviews and meta-analyses [3, 18, 25, 26], inhibitory control is decreased in patients with BED at least in comparison with normal weight controls. However, evidence in comparison with obese controls is heterogenous, especially for the SST [25, 26]. Moreover, the eSST considered negative emotions, but did not take disorder-specific features like food into account. Thus, this eSST might not address the disorder-related deficits in inhibitory control that have been found in patients with BED in other studies [3, 25, 26]. Additionally, anger might not have been the appropriate emotion to decrease inhibitory control especially in patients with BED, although previous evidence suggests this role of anger in BED [11, 12]. For instance, the patients with BED in our study did not show increased trait anger in comparison with the control groups. Though Zeeck et al. [11] reported that anger was most often preceding binge eating episodes, they also reported that the relationship between the desire to eat and binge eating was highest, when patients feel lonely, disgusting, exhausted or ashamed. Likewise, a current review delivers evidence especially for shame as a trigger for binge eating [40]. Another explanation could be that the simple presentation of angry facial expressions did not induce (enough) anger in the participants and thus, did not affect inhibitory control. Results from the mood ratings before and after the eSST support this assumption as mood decreased after the eSST, but was still rated as highly positive. As all participants were able to recognize the presented emotions very well, especially regarding angry and neutral faces where no group difference regarding emotion recognition emerged, and as there was no correlation between emotion recognition and eSST, a deficit in emotion recognition might not be a suitable explanation as well. Recently, Gupta and colleagues investigated the impact of anger in healthy participants by using a modified version of the SST by presenting emotional faces as stop signals, i.e. angry versus happy faces [29, 41]. However, they report conflicting findings on the impact of angry stimuli: Whereas Gupta and Singh [41] conclude that angry faces facilitate response inhibition in comparison with happy faces due to a freezing effect, Pandey and Gupta [29] state that angry faces impair response inhibition as they capture attention and that other, more evolutionary threatening stimuli might elicit cognitive freezing. These general population studies deliver interesting insights and might explain the general effect of anger versus neutral stimuli in all participants regardless of BMI and eating behavior. However, further research is needed to disentangle these possible explanations of the emotional impact on inhibitory control by neuroimaging studies.

Regarding correlational analyses, results have been sobering as well. Behavioral parameters from the eSST did not correlate with any of the proposed related variables, neither with eating disorder pathology, nor with negative urgency, trait anger or emotion regulation. Though self-reports and laboratory tasks are often not related to each other, especially concerning impulsivity concepts [21], and though the SST is well validated to measure inhibitory control, it might be that the eSST does not assess inhibitory control in conjunction with negative urgency. Further, it might be that the perception of emotional stimuli is not problematic for patients with BED or that angry stimuli induce rather anxious and not angry feelings. In the current sample, negative urgency and perseverance were increased in patients with BED, whereas regulation of negative emotions was decreased, so that patients with BED might rather show decreased inhibitory control while they experience angry emotions themselves. Thus, it might be more promising to investigate the interplay of negative emotions and inhibitory control by inducing anger versus neutral mood with a mood induction technique and present food as disorder-specific stimuli in an inhibitory control task. In this case, disorder-related as well as emotional factors would have been considered. However, mood induction techniques are not standardized and not easy to apply [42, 43].

With regard to the limitations of this study, we have to clearly state that this is a pilot study with a small sample size and smaller effects could be undetected due to poor statistical power. Further, even if not correlated with the outcomes of the eSST, the NW-CG reported a somewhat higher positive mood and lower depression scores in comparison with the patients with BED. Moreover, social emotional stimuli that are not disorder-related might not trigger impulsive reactions in patients with BED.

## Conclusions and perspectives

In sum, the current pilot study did not deliver evidence for decreased inhibitory control towards angry stimuli in patients with BED as all participants showed decreased inhibitory control in the angry versus neutral condition. It might be that this is due to a more general and not disorder-related effect, representing the conjunction of inhibitory control and anger as discussed by Gupta and colleagues [29, 41]. Nevertheless, dysfunctional emotion regulation seems a promising underlying mechanism for binge eating [5]. For further studies, it might be more fruitful to investigate the interplay of several discrete emotions and impulsive behavior in a more naturalistic setting, e.g., by ecological momentary assessments to identify those emotions that are strongest related to binge eating episodes. Studies in this direction have been conducted already some decades ago [8], but with the current technological opportunities, it might be possible to investigate the exact time course of binge eating in relation to mood in details.

## Data Availability

The datasets used and/or analyzed during the current study are available from the corresponding author on reasonable request.

## Abbreviations

BDI-II: Becks’ depression inventory II
BED: Binge eating disorder
BMI-CG: BMI-matched overweight and obese control participants
DSM-5: Diagnostic and statistical manual of mental disorders: 5th edition
EDE-Q: Eating disorder examination questionnaire
eSST: Emotional stop signal task
NMR-SF: Generalized expectancies for negative mood regulation
NW-CG: Normal-weight healthy control participants
SAM: Self-assessment manikins
SCID-I: Structured clinical interview for mental disorders
SSRT: Stop signal reaction time
STAXI-2: State-trait-anger-inventory-2
UPPS: UPPS impulsive behavior scale
VERT-K: Vienna emotion recognition task
